# Transperitoneal radical nephroureterectomy is associated with worse disease progression than retroperitoneal radical nephroureterectomy in patients with upper urinary tract urothelial carcinoma

**DOI:** 10.1038/s41598-019-42739-0

**Published:** 2019-04-18

**Authors:** Tae Heon Kim, Yoon Seok Suh, Hwang Gyun Jeon, Byong Chang Jeong, Seong Il Seo, Seong Soo Jeon, Hyun Moo Lee, Han Yong Choi, Hyun Hwan Sung

**Affiliations:** 1 Department of Urology, CHA Bundang Medical Center, CHA University, Seongnam, Republic of Korea; 2 Department of Urology, Samsung Medical Center, Sungkyunkwan University School of Medicine, Seoul, Republic of Korea; 3 Department of Urology, Kangbuk Samsung Hospital, Sungkyunkwan University School of Medicine, Seoul, Republic of Korea

**Keywords:** Ureter, Urological manifestations

## Abstract

This study aimed to compare the oncologic outcomes between retroperitoneal radical nephroureterectomy (rRNU) and transperitoneal radical nephroureterectomy (tRNU) for the treatment of patients with upper urinary tract urothelial carcinoma (UTUC). Medical records of 743 eligible patients who underwent rRNU or tRNU between 1995 and 2015 were reviewed retrospectively. Progression-free survival (PFS), cancer-specific survival (CSS), and overall survival (OS) were compared according to the surgical approach using the Kaplan–Meier method. Predictors of PFS, CSS, and OS were analyzed with a multivariable Cox regression model. Overall, 620 (83.4%) and 123 (16.6%) patients were treated with rRNU and tRNU, respectively. Five-year CSS and OS rates were equivalent between rRNU and tRNU groups, but 5-year PFS was lower in the tRNU group than the rRNU group (*P* = 0.005). When patients were stratified by pathological T stage, PFS was significantly different between the two groups in favor of the rRNU group for both organ-confined disease (pTis/pTa/pT1/T2) (*P* = 0.022) and locally advanced disease (pT3/pT4) (*P* = 0.039). However, no significant differences in CSS or OS was observed when comparing the two surgical types in patients with organ-confined disease (*P* = 0.336 and *P* = 0.851) or patients with locally advanced disease (*P* = 0.499 and *P* = 0.278). tRNU was a significant predictor of PFS (hazard ratio = 1.54; *P* = 0.023), but not CSS or OS. Our findings indicate that the rRNU approach resulted in better PFS than the tRNU approach in patients with UTUC.

## Introduction

The prevalence of upper urinary tract urothelial carcinoma (UTUC) has increased gradually, although it remains a rare disease that accounts for 5–10% of all urothelial carcinomas^1^. Radical nephroureterectomy (RNU) with bladder cuff excision is considered the gold standard for treating patients with non-metastatic UTUC^2^. Historically, RNU has been performed with an open approach, followed by a laparoscopic approach^3^. Advances in techniques, technology, and instruments have enabled less invasive laparoscopic RNU, which is increasingly being performed as a minimally invasive approach^4–6^. Both open and laparoscopic RNU can be performed via transperitoneal or retroperitoneal approaches, and each one has its own advantages and limitations. The advantage of the transperitoneal approach is the larger available working space and readily identifiable anatomic landmarks. In contrast, the retroperitoneal approach enables direct access to the renal artery and the kidney, and avoidance of bowel mobilization. Consistent with this, one study reported that the retroperitoneal approach was associated with faster recovery of bowel function and a shorter hospital stay than the transperitoneal approach^7^. However, no definitive conclusions regarding the oncologic outcomes of the two different surgical approaches have been reached to date. To clarify this issue, we evaluated and compared oncologic outcomes between transperitoneal and retroperitoneal approaches in patients who underwent RNU for UTUC.

## Materials and Methods

### Study population

We identified 820 consecutive patients who underwent RNU between February 1995 and December 2015 at our institution. After excluding patients with a benign mass or other malignancies on final pathology after RNU, history of radical cystectomy for invasive bladder cancer, bilateral UTUC, or no follow-up data, 743 patients were included in the analysis. Perioperative clinicopathological data including age, sex, body mass index, American Society of Anesthesiologists score, location of tumor, tumor multifocality, tumor size, surgical approach technique, type of bladder cuff excision, pathological T stage, tumor grade, carcinoma *in situ*, lymphovascular invasion (LVI), lymph node status, surgical margin status, follow-up duration, disease progression, and survival information were obtained from chart review. This study was approved by our Institutional Review Board of Samsung Medical Center (IRB approval number: 2016-10-100) and the requirement for written informed consent was waived.

### Study design

The medical records of 743 patients were reviewed retrospectively. The surgical approach method (retroperitoneal or transperitoneal) and technique (open or laparoscopic) that were used were determined by considering the clinical status of the patient and by the surgeon’s discretion after thorough and detailed consultation with the patient. Bladder cuff excision was classified as transvesical or extravesical. Regional lymph node dissection was performed if enlarged (≥1 cm) lymph nodes were present on preoperative computed tomography (CT) or magnetic resonance imaging (MRI) or were detected in the operative field. The extent of LND was usually determined by the surgeons based on preoperative image work-up, tumor location, and patient characteristics^8^. Detailed descriptions of RNU with bladder cuff excision have been provided in earlier publications^9,10^.

Although postoperative follow-up was not standardized because of the retrospective nature of this study, patients were generally followed-up every 3 months for the first year after surgery, every 6 months from the second to the fifth years, and annually thereafter. Urine cytology, cystoscopic examination, and routine checkups that included history taking, blood tests, and physical examination occurred at each visit. Imaging evaluations using CT or MRI were performed every 6 months for the first 5 years, then annually thereafter. Bone scans and chest CT were evaluated if clinically indicated.

### Outcome assessments

Patients were divided into two groups according to RNU surgical approach method: retroperitoneal RNU (rRNU) or transperitoneal RNU (tRNU). Clinical and pathological variables were compared between these two groups. Oncologic outcomes, including progression-free survival (PFS), cancer-specific survival (CSS), and overall survival (OS), were evaluated. Disease progression was defined as tumor relapse in the operative field or regional lymph nodes or distant metastasis on image work-up by CT or MRI that was not found in preoperative evaluations. The regional lymph node template of Kondo *et al*. was used for CT or MRI review for recurrence at lymph node sites^8^. Visceral metastasis was defined as metastasis to internal organs including the liver, lungs, and body cavity such as the pleura and peritoneum. Intraperitoneal space metastasis was defined as metastasis to organs inside the peritoneum. To analyze the effect of time since operation on oncological outcomes, patients were divided into quintiles according to serial case number.

### Statistical analysis

Medians and interquartile ranges (IQRs) were used to describe continuous variables, and frequencies and percentages were used for categorical variables. The Shapiro-Wilk normality test was used to investigate if continuous variables followed a normal distribution. The Mann-Whitney *U*-test was used to compare differences in continuous variables between the two groups. Categorical variables were compared using either Pearson’s chi-square test or linear-by-linear association. Fisher’ exact test was also used when appropriate. Kaplan–Meier curves were constructed to illustrate PFS, CSS, and OS according to surgical approach (rRNU versus tRNU). The Kaplan-Meier method was used to evaluate the influence of surgical approach method on PFS, CSS, and OS in the entire study cohort, pathological T stage subgroups, and the laparoscopic RNU subgroup. The log-rank test was used to assess differences. Prognostic factors for PFS, CSS, and OS were also analyzed using the multivariable Cox proportional hazard method. All statistical analyses were performed using SPSS^®^ software for Windows, version 21.0 (IBM Corp., Armonk, NY). A *P*-value < 0.05 was considered statistically significant, and all statistical tests were two-sided.

## Results

### Baseline characteristics

Clinicopathological characteristics according to surgical approach are summarized in Table 1. Overall, median age was 65.0 (IQR, 57.0–73.0) years, and 73.6% (547/743) of patients were male. Median follow-up duration was 50.0 (IQR, 26.2–95.2) months. Among 743 patients, 620 (83.4%) underwent rRNU and 123 (16.6%) underwent tRNU. There was no significant difference between the two groups with regard to age, sex, body mass index, American Society of Anesthesiologists score, location of tumor, tumor multifocality, pathological T stage, tumor grade, carcinoma *in situ*, LVI, lymph node status, or surgical margin status. However, a laparoscopic approach was used more often in the tRNU group (*P* < 0.001), and this group of patients also underwent more extravesical excision for distal ureter management (*P* = 0.026), and had a shorter follow-up duration (*P* = 0.034) than the rRNU group.

**Table 1:** Clinicopathological characteristics of the 743 patients who underwent radical nephroureterectomy by either a retroperitoneal or transperitoneal approach.

	All patients (*n* = 743)	Retroperitoneal approach (*n* = 620)	Transperitoneal approach (*n* = 123)	*P* value
Age, years,	65.0 (57.0–73.0)	65.0 (57.0–72.0)	67.0 (58.0–75.0)	0.235
Sex, male	547 (73.6)	454 (73.2)	93 (75.6)	0.584
Body mass index, kg/m^2^	24.1 (22.3–25.9)	24.1 (22.3–25.9)	24.1 (22.0–26.2)	0.957
ASA score ≥ II, n (%)	527 (70.9)	438 (70.6)	89 (72.4)	0.702
Case number (quintile)				<0.001
1–149		139 (93.3)	10 (6.7)	
150–298		128 (85.9)	21 (14.1)	
299–447		121 (81.2)	28 (18.8)	
448–596		120 (80.5)	29 (19.5)	
597–743		112 (76.2)	35 (23.8)	
Tumor location				0.179
Renal pelvis	366 (49.3)	311 (50.2)	55 (44.7)	
Ureter	296 (39.8)	245 (39.5)	51 (41.5)	
Both renal pelvis and ureter	81 (10.9)	64 (10.3)	17 (13.8)	
Multifocal tumor	178 (24.0)	145 (23.4)	33 (26.8)	0.414
Approach technique				<0.001
Open	268 (36.1)	260 (41.9)	8 (6.5)	
Laparoscopy	475 (63.9)	360 (58.1)	115 (93.5)	
Type of bladder cuff excision, n (%)				0.026
Transvesical	394 (53.0)	340 (54.8)	54 (43.9)	
Extravesical	349 (47.0)	280 (45.2)	69 (56.1)	
Pathological T stage				0.865
pTis/Ta	121 (16.3)	100 (16.1)	21 (17.1)	
pT1	179 (24.1)	149 (24.0)	30 (24.4)	
pT2	131 (17.6)	111 (17.9)	20 (16.3)	
pT3/T4	312 (42.0)	260 (41.9)	52 (42.3)	
Tumor grade				0.937
I/II	394 (53.0)	329 (53.1)	65 (52.8)	
III	335 (45.1)	279 (45.0)	56 (45.5)	
Missing/unknown	14 (1.9)	12 (1.9)	2 (1.6)	
Pathologic N stage, n (%)				0.136
pNx/pN0	673 (90.6)	566 (91.3)	107 (87.0)	
≥pN1	70 (9.4)	54 (8.7)	16 (13.0)	
Concomitant CIS, positive	77 (10.4)	66 (10.6)	11 (8.9)	0.572
Concomitant LVI, positive	133 (17.9)	109 (17.9)	24 (19.5)	0.610
Surgical margin, positive	34 (4.6)	30 (4.8)	4 (3.3)	0.442
Follow-up duration, months	50.0 (26.2–95.2)	53.1 (27.3–96.8)	43.4 (25.2–70.0)	0.034

The Shapiro-Wilk normality test was used to investigate the normal distribution of continuous variables. All values are given as medians (interquartile ranges) or numbers (%) of patients. ASA = American Society of Anesthesiologists; CIS = carcinoma *in situ*; LVI = lymphovascular invasion.

### Oncologic outcomes

**Figure 1:**
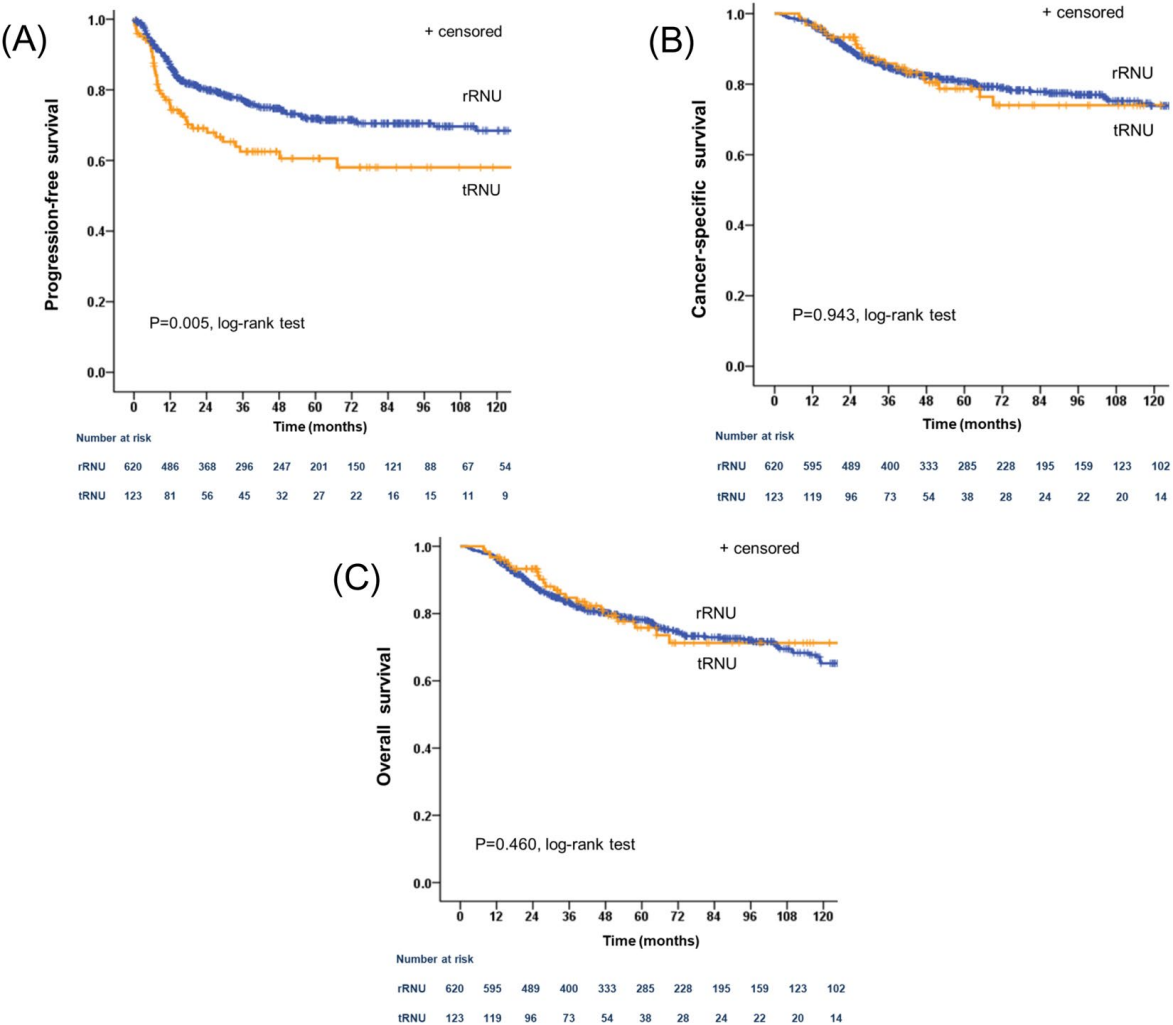
Cumulative survival of 743 patients after radical nephroureterectomy for upper tract urothelial carcinoma according to surgical approach method. (**A**) Progression-free survival; (**B**) cancer-specific survival; (**C**) overall survival. rRNU = retroperitoneal radical nephroureterectomy; tRNU = transperitoneal radical nephroureterectomy.

At last follow-up, there were 26.0% (193/743) cases of disease progression, including 24.4% (151/620) in the rRNU group and 34.1% (42/123) in the tRNU group. Locations of progression after RNU are summarized in Table 2. No significant difference was noted in tumor relapse in the operative field. However, tumor relapse in regional lymph nodes (*P* = 0.002) and distant metastasis (*P* = 0.004) were more frequent in the tRNU group than the rRNU group. Progression to the intraperitoneal space was more common in the tRNU group than the rRNU group (14.6% versus 4.0%, respectively, P < 0.001). Five-year PFS rates were 71.9% and 60.6% for patients in the rRNU and tRNU groups, respectively, indicating that patients in tRNU group had a poorer PFS than those in the rRNU group (*P* = 0.005; Fig. 1A). Among the 186 (25.0%) patients who died during follow-up, 146 (19.7%) deaths were directly related to UTUC. Five-year CSS rates were 80.8% and 78.7% in the rRNU and tRNU groups, respectively (Fig. 1B). Five-year OS rates were 78.2% and 75.8% in the rRNU and tRNU groups, respectively (Fig. 1C). No significant difference was observed in CSS and OS when comparing rRNU versus tRNU groups (P = 0.943 and P = 0.460, respectively).

**Table 2:** Incidence and location of progression after radical nephroureterectomy according to surgical approach.

	All patients (*n* ll743)	Retroperitoneal approach (*n* et620)	Transperitoneal approach (*n* ra123)	*P* value
Progression, n (%)	193 (26.0)	151 (24.4)	42 (34.1)	
Location of progression, n (%)				
Operative field	42 (5.7)	37 (6.0)	5 (4.1)	0.404
Regional lymph node	113 (15.2)	83 (13.4)	30 (24.4)	0.002
Distant metastasis	100 (13.5)	75 (12.1)	25 (20.3)	0.015
Visceral metastasis	89 (12.0)	68 (11.0)	21 (17.1)	0.057
Lung	49	36	13	
Liver	4	3	1	
Pancreas	2	2	0	
Intestine	1	1	0	
Peritoneum	1	0	1	
Muscle	1	1	0	
Multiple sites	31	25	6	
Distant lymph node	18 (2.4)	6 (1.0)	12 (9.8)	<0.001
Bone metastasis	17 (2.3)	16 (2.6)	1 (0.8)	0.333
Intraperitoneal space	43 (5.8)	25 (4.0)	18 (14.6)	<0.001

**Figure 2:**
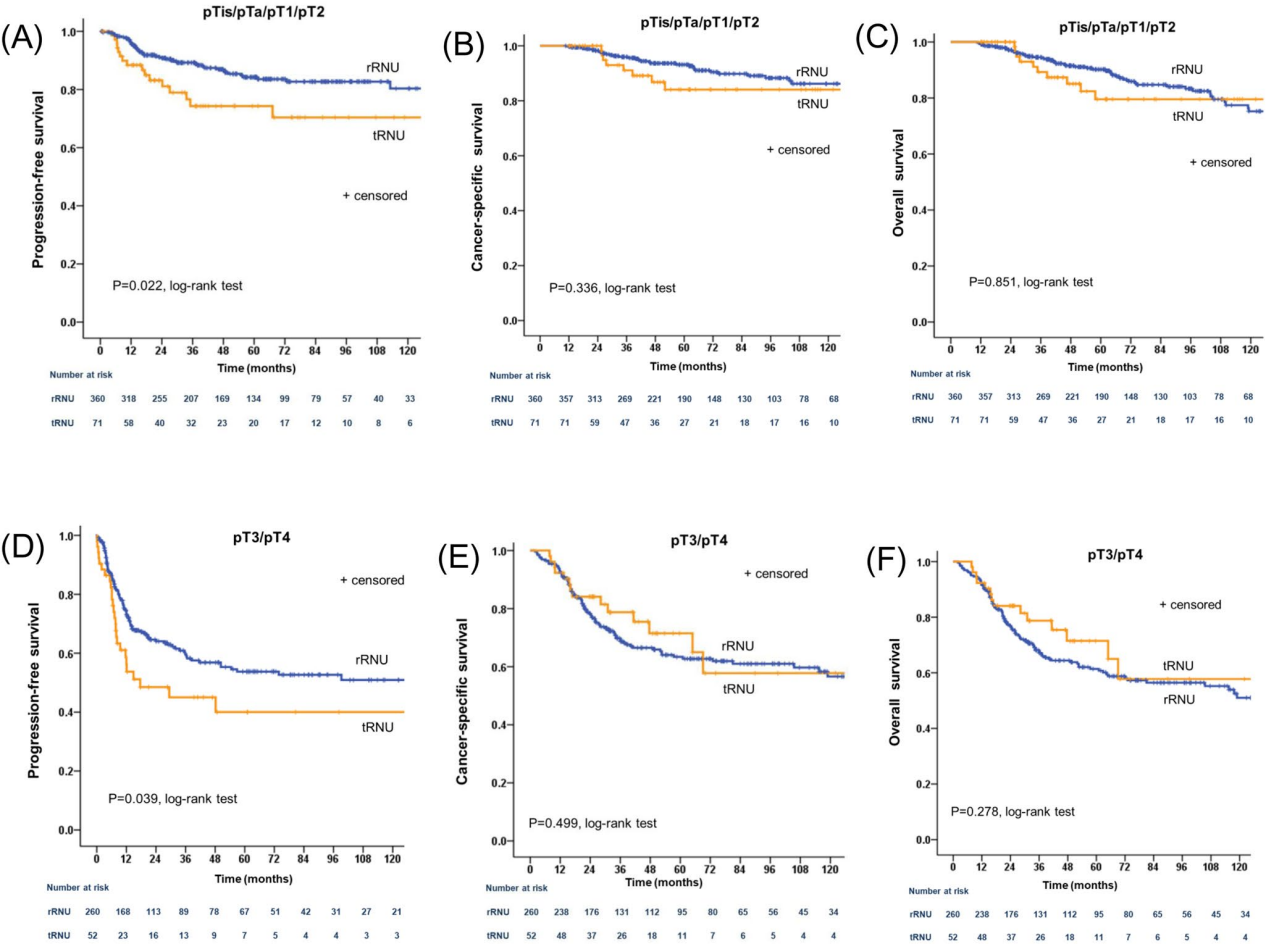
Cumulative survival of subgroups stratified by pathological T state according to surgical approach method. (**A**) Progression-free survival in patients with pTis/pTa/pT1/pT2; (**B**) cancer-specific survival in patients with pTis/pTa/pT1/pT2; (**C**) overall survival in patients with pTis/pTa/pT1/pT2; (**D**) progression-free survival in patients with pT3/pT4; (**E**) cancer-specific survival in patients with pT3/pT4; (**F**) overall survival in patients with pT3/pT4. rRNU = retroperitoneal radical nephroureterectomy; tRNU = transperitoneal radical nephroureterectomy.

**Figure 3:**
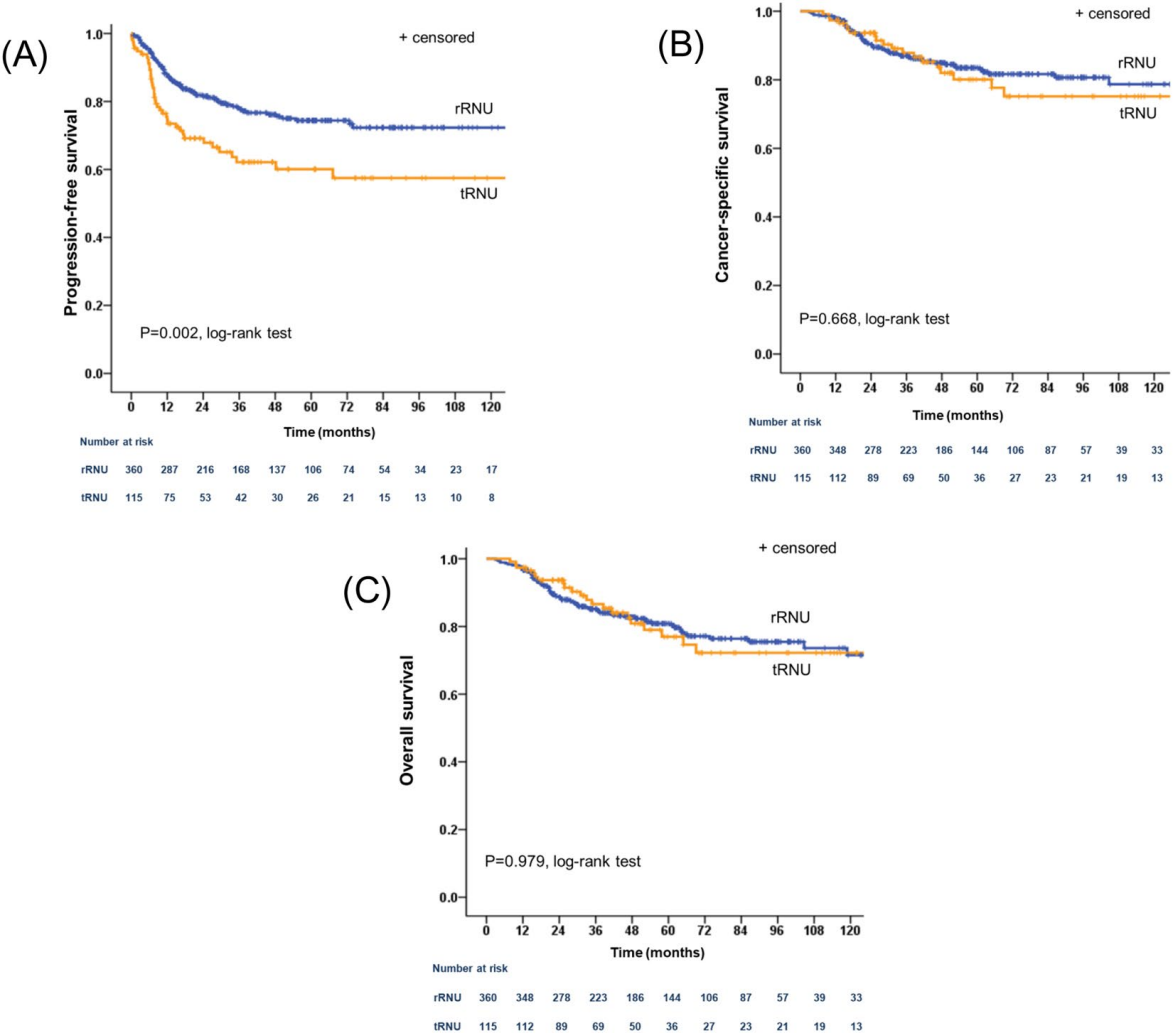
Cumulative survival of 475 patients who underwent laparoscopic radical nephroureterectomy for upper tract urothelial carcinoma according to surgical approach method. (**A**) Progression-free survival; (**B**) cancer-specific survival; (**C**) overall survival. rRNU = retroperitoneal radical nephroureterectomy; tRNU = transperitoneal radical nephroureterectomy.

When patients were stratified by pathological T stage, PFS was significantly different between the two groups in favor of the rRNU group for both organ-confined disease (pTis/pTa/pT1/T2) (*P* = 0.022; Fig. 2A) and locally advanced disease (pT3/pT4) (*P* = 0.039; Fig. 2D). However, no significant differences in CSS and OS were observed when comparing the two surgical approaches in patients with organ-confined disease (pTis/pTa/pT1/T2) (*P* = 0.336; Fig. 2B and *P* = 0.851; Fig. 2C, respectively) or patients with locally advanced disease (pT3/pT4) (*P* = 0.499; Fig. 2E and *P* = 0.278; Fig. 2F, respectively). Subgroup analysis of patients who underwent laparoscopic RNU also revealed that patients in the rRNU group had a better PFS than those in the tRNU group (*P* = 0.002; Fig. 3A), but the CSS and OS of the two groups were similar (*P* = 0.668; Fig. 3B and *P* = 0.979; Fig. 3C, respectively).

### Prognostic factors for progression, death from UTCS, and all-cause death after RNU

Table 3 presents the results of the multivariable Cox regression analysis examining predictors of progression, death from UTUC, and all-cause death in the entire study cohort. tRNU was significantly correlated with increased risk of progression (hazard ratio [HR] = 1.54; *P* = 0.023), but tRNU was not an independent predictor of death from UTUC (HR = 1.12, *P* = 0.650) or all-cause death (HR = 0.96, *P* = 0.880). Female sex (HR = 1.38; *P* = 0.049) and ureter-involved tumors (HR = 1.49; *P* = 0.017) were significantly associated with progression. An extravesical approach as the method of bladder cuff excision was a significant independent predictor of all-cause death (HR = 1.49; *P* = 0.034). Tumor grade was independently associated with progression (HR = 1.84; *P* = 0.001) and death from UTUC (HR = 1.52; *P* = 0.036). Positive surgical margin status was significantly correlated with increased death from UTUC (HR = 2.29; *P* = 0.007) and all-cause death (HR = 2.38; *P* = 0.002). Age, quintile according to serial case number, pathological T state, pathological N stage, and presence of concomitant LVI were significantly associated with progression, death from UTUC, and all-cause death.

**Table 3:** Multivariable Cox proportional hazard regression analyses to predict progression, death from upper tract urothelial carcinoma, and all-cause death in 743 patients with upper tract urothelial carcinoma treated with radical nephroureterectomy.

	Progression			Death from UTUC			All-cause death		
	HR	95% CI	*P* value	HR	95% CI	*P* value	HR	95% CI	*P* value
Age (continuous)	1.02	1.00-1.03	0.022	1.04	1.02–1.06	<0.001	1.04	1.03–1.06	<0.001
Sex									
Male	Reference			Reference			Reference		
Female	1.38	1.00–1.89.	0.049	1.24	0.86–1.79	0.245	1.09	0.78–1.52	0.618
ASA									
≤1	Reference			Reference			Reference		
≥2	0.80	0.57–1.11	0.181	1.15	0.78–1.69	0.479	1.08	0.77–1.51	0.667
Case number									
1–149	Reference			Reference			Reference		
150–298	0.60	0.38–0.94	0.027	0.47	0.30–0.75	0.001	0.48	0.32–0.72	<0.001
299–447	0.83	0.51–1.35	0.457	0.46	0.26–0.81	0.007	0.48	0.29–0.79	0.004
448–596	0.76	0.46–1.25	0.272.	0.21	0.11–0.41	<0.001	0.23	0.13–0.43	<0.001
597–743	0.56	0.32–0.97	0.039	0.08	0.03–0.23	<0.001	0.08	0.03–0.22	<0.001
Tumor location									
Only renal pelvis	Reference			Reference			Reference		
Ureter involvement	1.49	1.08–2.07	0.017	1.34	0.91–1.96	0.141	1.29	0.92–1.82	0.136
Multifocal tumor									
No	Reference			Reference			Reference		
Yes	1.31	0.94–1.83	0.108	1.25	0.85–1.83	0.257	1.18	0.84–1.66	0.352
Approach method									
Retroperitoneal	Reference			Reference			Reference		
Transperitoneal	1.54	1.06–2.25	0.023	1.12	0.68–1.87	0.650	0.96	0.60–1.54	0.880
Approach technique									
Open	Reference			Reference			Reference		
Laparoscopic	1.10	0.78–1.54	0.598	0.98	0.67–1.46	0.983	0.91	0.64–1.28	0.581
Type of bladder cuff excision									
Transvesical	Reference			Reference			Reference		
Extravesical	1.22	0.87–1.70	0.248	1.44	0.96–2.17	0.082	1.49	1.03–2.14	0.034
Pathological T stage									
pTis/pTa/pT1/pT2	Reference			Reference			Reference		
pT3/pT4	2.50	1.77–3.52	<0.001	3.19	2.11–4.82	<0.001	2.32	1.63–3.29	<0.001
Tumor grade									
I/II	Reference			Reference			Reference		
III	1.84	1.30–2.61	0.001	1.52	1.03–2.25	0.036	1.37	0.97–1.92	0.076
Pathological N stage									
pNx/pN0	Reference			Reference			Reference		
≥pN1	1.91	1.28–2.86	0.002	2.65	1.72–4.10	<0.001	2.69	1.79–4.04	<0.001
Concomitant CIS									
No	Reference			Reference			Reference		
Yes	0.62	0.37–1.05	0.074	0.63	0.34–1.14	0.126	0.71	0.42–1.20	0.205
Concomitant LVI									
No	Reference			Reference			Reference		
Yes	1.90	1.34–2.70	<0.001	1.58	1.06–2.36	0.024	1.55	1.07–2.23	0.019
Surgical margin									
negative	Reference			Reference			Reference		
positive	1.48	0.85–2.59	0.171	2.29	1.25–4.20	0.007	2.38	1.38–4.10	0.002

UTUC = upper urinary tract urothelial carcinoma; HR = hazard ratio; CI = confidence interval; ASA = American Society of Anesthesiologists; CIS = carcinoma *in situ*; LVI = lymphovascular invasion.

## Discussion

In the present study, we evaluated the associations between surgical approach (rRNU and tRNU) and oncologic outcomes in patients with UTUC. Our study demonstrated that 5-year CSS and OS rates were equivalent between the rRNU and tRNU groups, but that 5-year PFS was lower in the tRNU group than the rRNU group (Fig. 1). These results were consistent in pathological T stage subgroup analysis, where a rRNU approach was related to better PFS than a tRNU approach for both pTis/pTa/pT1/T2 tumors and pT3/pT4 tumors. The 5-year PFS for pTis/pTa/pT1/T2 tumors was 84.2% in the rRNU group and 74.3% in the tRNU group (*P* = 0.022), while the 5-year PFS for pT3/pT4 tumors was 53.7% in the rRNU group and 40.0% in the tRNU group (*P* = 0.039). In patients treated with laparoscopic RNU, there was also strong evidence of a PFS advantage in the rRNU group, with a 5-year PFS of 74.4% as compared with 60.1% in the tRNU group (*P* = 0.002). Furthermore, tRNU was a significant predictor of PFS, but not CSS or OS.

A transperitoneal or retroperitoneal approach can be used for either open or laparoscopic RNU. In general, a better anatomical orientation and larger operational field can be achieved with tRNU. With rRNU, direct access to the kidney without mobilization of the bowel and intraperitoneal organs near the kidney and ureter can reduce complications involving the intraperitoneal organs^7^. However, the impacts of these approaches on oncologic outcomes are not yet well known. To the best of our knowledge, studies that have examined differences in oncologic outcomes between these two approaches in patients with RNU are rare. A retrospective study by Liu *et al.*^7^ compared 34 laparoscopic rRNU patients with 34 laparoscopic tRNU patients. They found that both laparoscopic rRNU and tRNU were safe and effective approaches for management of UTUC with comparable perioperative outcomes, but time to first oral intake and hospital discharge were faster in the rRNU group than the tRNU group.

In the present study, we evaluated the associations between surgical approach method (rRNU and tRNU) and oncologic outcomes in a large cohort of patients with UTUC who underwent either open or laparoscopic RNU. In our study, the proportion of laparoscopic RNUs was significantly higher in the tRNU group than the rRNU group. Although several studies have been conducted to compare the oncologic outcomes of open RNU and laparoscopic RNU in patients with UTUC, findings have been conflicting, and there is no accepted conclusion as to which surgical approach technique (open or laparoscopic) is more beneficial for patients with UTUC^11–13^. A meta-analysis of 21 publications by Ni *et al*.^14^ reported no significant differences in recurrence-free survival or OS between open RNU and laparoscopic RNU. We also found that surgical approach technique was not related to PFS, CSS, or OS (Table 3). The type of bladder cuffing performed was not evenly distributed between the rRNU and tRNU groups in our cohort. However, no consensus exists on the optimal bladder cuffing approach in terms of oncologic outcome^9,15,16^. In a retrospective study of 2,681 patients who underwent RNU by extravesical, transvesical, or endoscopic approaches, no differences were found in non-bladder recurrence or survival among the three groups^17^. When we analyzed the location of progression after RNU, we found that recurrence at regional lymph nodes, distant lymph node metastasis, and intraperitoneal metastasis rate were significantly higher in the tRNU group than the rRNU group. These results might to be related to early ligation of the renal pedicle and ureter during rRNU. The rRNU procedure enables earlier exposure of the kidney and ureter compared to the tRNU procedure. Early ligation of the renal pedicle before manipulation of the kidney or ureter can minimize migration of tumor cells to the bloodstream or urinary system. Another explanation for our results is that exposure of the intraperitoneal space during the tRNU procedure provides a chance for spillage of tumor cells directly into the intraperitoneal space. In addition, in the rRNU procedure, bladder cuff excision is performed through an extraperitoneal approach, which may reduce the possibility of intraperitoneal tumor cell spillage and contribute to better disease progression outcomes in the rRNU group than the tRNU group. Of note, superiority of PFS in the rRNU group was consistent in subgroup analysis by both tumor stage and laparoscopic RNU group, but no significant difference was found in CSS or OS between the rRNU and tRNU groups (Figs 2 and 3). This discrepancy between survival indexes might have resulted from the relatively short follow-up duration of our study. This issue is expected to be clarified in a future study with a longer follow-up.

Several studies have reported prognostic factors for UTUC. Tumor stage and lymph node status are the most important predictors of survival^18^. Recent studies have demonstrated that high tumor grade, age, and LVI are independent prognostic factors for UTUC^19–23^. Most prognostic factors of disease progression in our study were consistent with those reported in previous studies. Female patients and ureteral involvement of the tumor were associated with progression after RNU in our study. A transperitoneal approach was also a prognostic factor for disease progression after RNU.

Limitations of this study include its retrospective nature. In addition, because this study was conducted with data collected from a single institution, selection bias might have occurred. Moreover, a selection bias might also result from the surgeon effect. The choice of surgical approach was mainly determined by the surgeon’s preference in addition to the patient’s characteristics. Thus, the distribution among tRNU and rRNU was different, and this uneven distribution could have affected the observed differences. Differences in pathological characteristics and use of adjuvant chemotherapy among patients are other potential confounding factors. In this study, we did not assess the status of neoadjuvant or adjuvant chemotherapy of each patient. The impact of chemotherapy for UTUC is still under debate, especially neoadjuvant chemotherapy. Furthermore, some reports showed that receipt of adjuvant chemotherapy was not related to disease progression, death from UTUC, and all-cause death^24^. Management of regional lymph nodes differed among patients because of the retrospective design and absence of a standardized template for lymph node dissection. The therapeutic benefits of nodal status and lymph node dissection for disease-free and cancer-free survival remain controversial and need to be clarified^25,26^. Although our institution followed recommendations and institutional protocols^27^, there was a lack of standardization of the follow-up program. It also might affect evaluation of the real impact of surgical approach on oncologic outcomes. Nevertheless, despite these limitations, our study results are highly relevant as they delineate the effect of surgical approach method on the oncologic outcome of RNU in patients with UTUC.

## Conclusion

In conclusion, the rRNU approach resulted in better oncologic control of disease progression than the tRNU approach in patients with UTUC, and surgical approach method was a significant predictive factor of PFS. No significant difference in CSS or OS was observed between the rRNU and tRNU groups.
